# How does teacher-perceived principal leadership affect teacher self-efficacy between different teaching experiences through collaboration in China? A multilevel structural equation model analysis based on threshold

**DOI:** 10.3389/fpsyg.2022.933838

**Published:** 2022-08-25

**Authors:** Zhiyong Xie, Rongxiu Wu, Hongyun Liu, Jian Liu

**Affiliations:** ^1^College of Teacher Education, South China Normal University, Guangzhou, China; ^2^Neag School of Education, University of Connecticut, Storrs, CT, United States; ^3^Beijing Key Laboratory of Applied Experimental Psychology, Faculty of Psychology, Beijing Normal University, Beijing, China; ^4^Collaborative Innovation Center of Assessment for Basic Education Quality, Beijing Normal University, Beijing, China

**Keywords:** teacher-perceived principal leadership, teacher collaboration, teaching, teaching experience threshold, teacher self-efficacy

## Abstract

Teacher self-efficacy is one of the most critical factors influencing Students’ learning outcomes. Studies have shown that teacher-perceived principal leadership, teacher collaboration, and teaching experience are the critical factor that affects teacher self-efficacy. However, little is known about the mechanisms behind this relationship. This study examined whether teacher collaboration would mediate the relationship between teacher-perceived principal leadership and teacher self-efficacy, and the moderating role of teaching experience in the mediating process. With an analysis of a dataset from 14,121 middle school teachers in China, this study first testified to the positive role that teacher-perceived principal leadership played in teacher self-efficacy. Furthermore, it revealed that teacher collaboration mediates this relationship and the mediated path was moderated by teaching experience. Finally, it also indicated that the threshold of teaching experience linking the teacher-perceived leadership with teacher self-efficacy was approximately in the third year, and their relationship was stronger when teaching experience was below the threshold. This study highlighted the mediating and moderating mechanisms linking the teacher-perceived principal leadership and teacher self-efficacy, which has important theoretical and practical implications for intervention and enhancement of teacher self-efficacy.

## Introduction

Teaching is an indispensable and irreplaceable factor impacting Students’ learning outcomes (Rockoff, 2004; Hattie, 2008). Effective teaching greatly improves Students’ academic achievement and narrows the gaps among students with various socioeconomic and cultural backgrounds (Odden et al., 2004; Akiba et al., 2007). The international community has paid increasing attention to teaching quality and teaching effectiveness in the past decades (Klassen et al., 2011). Among the factors that impact teaching quality and effectiveness, teacher self-efficacy, “a belief in their abilities to plan, organize, and carry out activities required to attain given educational goals” (Skaalvik and Skaalvik, 2007), has been found to play an important role in influencing teachers’ classroom performance and achieving satisfactory teaching results (Tschannen-Moran and Hoy, 2001; Bellibas and Liu, 2017). Considering the promoting effect of teacher self-efficacy on Students’ academic achievement, it is of critical value to explore the predictors of teacher self-efficacy. Rooted in the self-efficacy theory (Bandura, 1986), a body of extant research has indicated that principal leadership, teacher collaboration, and teachers’ teaching experience are the main predictors of teacher self-efficacy (Tschannen-Moran and Hoy, 2007; Avalos, 2011; deVries et al., 2013; Duyar et al., 2013; Fackler and Malmberg, 2016; Lambersky, 2016; Pfitzner-Eden, 2016; Bellibas and Liu, 2017; Li et al., 2019; Ma and Marion, 2021). Principals can promote positive teacher efficacy through direct supervision and engagement in instructional leadership activities (Duyar et al., 2013). Teachers’ collaboration refers to working with colleagues, which involves a series of activities such as exchanging feedback on teaching tasks, or participation in continuing professional development (CPD), which can cultivate a mutual learning environment and promote teachers’ teaching skills, and therefore contribute to Students’ academic achievement (deVries et al., 2013; Rutherford et al., 2017). The years that teachers have worked and their self-efficacy remain undecided as positive, negative, and no association have all been concluded, and therefore, there is a need for further investigation regarding their relationship (Li et al., 2019).

In present-day China, one of the most important activities of school principals is to organize novice teachers to pair up with experienced teachers to cooperate in teaching and research activities, so as to improve the teachers’ teaching skills and their self-efficacy afterward in the school (Li et al., 2019). The paired cooperation between novice and experienced teachers in teaching and research activities has gradually become the tradition of teachers’ professional development in China. With the popularity of this new format of teacher collaboration, there is a necessity to investigate the relationship between principal leadership, teacher collaboration, teaching experience, and teacher self-efficacy. Moreover, few studies have been conducted to explore the differences between novice and experienced teachers in the relationship. Furthermore, the trend of teachers’ professional development has been discussed in non-linear ways (Garner and Kaplan, 2021). With the complex dynamic systems (CDS) approach, they studied teachers’ professional development learning as a complex system, which can respond adaptively to internal and external changing conditions. With different years of teaching experience, teachers may exhibit differences in cognitive dissonance as well as when confronting conflicting beliefs, values, and practices.

Therefore, in our study, it was hypothesized that a non-linear relationship existed between teachers’ teaching experience and teacher self-efficacy and a threshold existed in dividing the notice and experienced teachers. With this hypothesis, it was expected to explore the differences between novice and experienced teachers in the relationship between principal leadership, teacher collaboration, teaching experience, and teacher self-efficacy, and hopefully could provide an in-depth nuanced understanding of the development of teacher self-efficacy and empirical data evidence from the Asian country to enrich teacher self-efficacy theory.

## Literature review

### Relationship between teacher-perceived principal leadership and teacher self-efficacy

Principal leadership refers to the ability that the school managers have in commanding, leading, and interacting with the team to achieve the school’s developmental goals (Bush and Glover, 2014). Modern educational management theory holds that effective principal leadership creates a positive school atmosphere through perception, behavior, and interaction related to the core driving force of teaching and learning (Blase, 1987; Leithwood, 1992; Hallinger et al., 2017; Liu and Hallinger, 2018). Principal leadership is a key factor in determining a school’s performance, which directly or indirectly affects teachers’ professional development and Students’ academic achievement (Leithwood, 1992; Dinham, 2007; Leithwood et al., 2010; Gumus et al., 2016; Bellibas and Liu, 2017). One important responsibility that school principals take in schools is to provide opportunities for teachers to develop teachers’ professional abilities. Teachers are often found to have stronger self-perceptions of principal leadership when they have more professional development opportunities organized by schools. At the same time, teachers who perceive higher levels of principal leadership are more supportive of schools’ development visions and are more likely to develop higher self-efficacies (Fackler and Malmberg, 2016; Lambersky, 2016; Gkolia et al., 2018; Liu and Hallinger, 2018). In this study, the term teacher-perceived principal leadership was used to refer to the teachers’ self-perception of principal leadership and it was hypothesized that a positive relationship existed between teacher-perceived principal leadership and teacher self-efficacy.

### The mediating role of teacher collaboration

As a professional practice of high interest, teacher collaboration plays a critical role in various teachers’ work, including instructional practice and professional learning (Goddard et al., 2007; Desimone, 2009; Chong and Kong, 2012; Muckenthaler et al., 2020). Schools are viewed as potential “communal organizations” characterized by “enhanced collegiality and collaboration,” within which collaboration may occur (Hausman and Goldring, 2001). Teacher collaboration is an essential part of teaching activities to establish and keep relationships among school staff (Chong and Kong, 2012; Muckenthaler et al., 2020).

According to Bandura (1997), teacher self-efficacy comes from four sources, which are mastery experiences, vicarious experiences, verbal persuasion, and emotional and physiological states. From this perspective, through cooperating in preparation for class, teaching, class evaluation, and teaching reflection, teachers not only develop mastery experiences, vicarious experiences, and verbal persuasion but also improve their confidence in teaching through continuous teaching cooperation and communication. Therefore, teacher collaboration provides a great opportunity for the development of teacher self-efficacy, and teacher activities which have teaching collaboration involved are more likely to develop their self-efficacy.

Furthermore, the higher the teacher perceives principal leadership in a school, the more cooperative teaching opportunities teachers can get and participate to improve teaching (Goddard et al., 2007). Therefore, it can be reasonably hypothesized that there exists a relationship between teacher-perceived principal leadership and teacher collaboration. When teachers participate in more cooperative teaching and research activities, teacher self-efficacy is more likely to develop. Studies have also shown that teacher collaboration has exerted a significant positive impact on teachers’ professional growth (Egodawatte et al., 2011; Vangrieken et al., 2015). Increased teacher collaboration was associated with a higher level of teacher self-efficacy (Shachar and Shmuelevitz, 1997; Yang, 2020). Vangrieken et al. (2015) concluded that teachers’ positive outcomes, including improvement in instruction, heightened efficacy, and improved professional knowledge are often documented. Therefore, it was hypothesized that teacher collaboration played a mediating role between teacher-perceived principal leadership and teacher self-efficacy in this study.

### The moderating role of teaching experience

Although teacher-perceived principal leadership is a positive factor of teacher self-efficacy through the intermediary role of teacher collaboration, this may not apply to all due to individual differences (Klassen and Chiu, 2010; Li et al., 2019). Teaching experience is the relevant experience accumulated by individuals engaged in teaching throughout the years. One source of teacher self-efficacy is teachers’ teaching experience (Bandura, 1986, 1997), which is closely related to teachers’ years of teaching. The longer the years of teaching, the more teaching experience they are to accumulate, and the higher their self-efficacy is. A positive relationship has been testified between teachers’ teaching experience and their self-efficacy (Prieto and Altmaier, 1994; Klassen and Chiu, 2010; Li et al., 2019).

Teachers’ professional careers can be divided into preservice and in-service phases, and they can be further divided into additional phases (Eros, 2011; Richter et al., 2011). Compared with experienced teachers with long years of teaching, novice teachers with short years of teaching may exhibit more obvious perceptions of principal leadership (Fantilli and McDougall, 2009; Bellibas and Liu, 2017; Mikser et al., 2020). The impact of teacher-perceived principal leadership on teacher self-efficacy may vary with teachers’ teaching experience. Novice teachers with short years of teaching experience need more communication and cooperation between teachers in the initial period of teaching while experienced teachers may not. Therefore, we can reasonably hypothesize that teacher collaboration is more likely to play a role in teacher self-efficacy for novice teachers than for experienced teachers with long years of teaching experience.

### The current study

**FIGURE 1:**
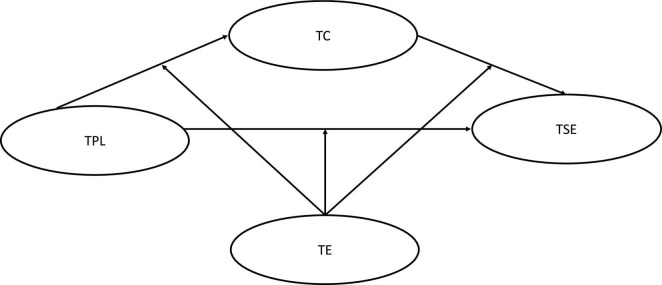
Structure of the proposed model. TPL, Teacher-perceived principal leadership; TC, Teacher collaboration; TE, Teaching experience; TSE, Teacher self-efficacy.

To further explore the nature of this relationship between teacher-perceived principal leadership and teacher self-efficacy, the current study examines a conceptual model (Figure 1) using a sample of Chinese high school teachers through a series of hypotheses. Specifically, we have made three hypotheses, as follows:

H1: Teacher-perceived principal leadership was positively and directly related to teacher self-efficacy.

H2: Teacher collaboration would play a mediating role in the relationship between teacher-perceived principal leadership and teacher self-efficacy. Teacher-perceived principal leadership would be positively related to teacher collaboration, which in turn would be positively associated with teacher self-efficacy.

H3: Teaching experience would moderate the direct and indirect relationship between teacher-perceived principal leadership and teacher self-efficacy through teacher collaboration.

In addition, this study divided teachers into novice and experienced through the non-linear relationship between teaching experience and teacher self-efficacy and explored the differences between novice and experienced teachers in the above model.

## Materials and methods

### Participant

The data we selected were from Region Education Assessment Project (REAP), a large-scale education assessment project in China. A two-stage stratified sample design was used to collect data from a province in the eastern area of China in 2018. According to the basic requirements of stratified sampling on sample size (Johnson and Christensen, 2019) and the need for a local education department, we randomly selected 90% of high schools in each city. It resulted in 139 high schools overall. Next, 50% of the second-grade teachers in high schools were randomly assigned to participate in the study, with a total of 14,121 teachers (male: 46.3%), whose ages ranged from 22 to 60 years (*M* = 38.93, *SD* = 8.17). Their academic degrees were specifically: associate (23.4%), bachelor (65.9%), and graduate (10.7%). These teachers were required to complete a survey including relevant demographic information (gender, age, educational background, and professional title), teacher-perceived principal leadership, teacher collaboration, teaching experience, and teacher self-efficacy information.

### Measures

#### Teacher-perceived principal leadership

TALIS2013 principal leadership scale (OECD, 2014) was modified to measure teacher-perceived principal leadership. The scale included five self-reported items (e.g., *Principal took actions to support collaboration among teachers to develop new teaching practices*) on a five-point Likert scale (1 = strongly disagree to 5 = strongly agree). Higher total scores indicate higher levels of teacher-perceived principal leadership. The goodness of fit is acceptable: χ^2^(3, 14,121) = 123.62, *p* < 0.001, RMSEA = 0.05, CFI = 0.99, TLI = 0.99, and SRMR = 0.003. The reliability McDonalds’ omega (ω) was 0.96 (>0.90) (Watkins, 2017; Hayes and Coutts, 2020) and therefore, considered acceptable.

#### Teacher collaboration

This study adopted the TALIS2013 teacher collaboration scale to measure teacher collaboration (OECD, 2014). In this scale, teachers reported their individual teaching collaboration activities with the questionnaire “On average, how often do you do the following in this school?” One sample item was “*Observe other teachers’ classes and provide feedback*.” Teachers’ responses consisted of the following options: 1 (never), 2 (1–2 times per month), 3 (3–5 times per month), 4 (6–9 times per month), and 5 (10 or more times per month). A higher score suggested a greater tendency for teacher collaboration. The goodness of fit is acceptable: χ^2^(1, 14,121) = 13.78, *p* < 0.001, RMSEA = 0.03, CFI = 1.00, TLI = 0.99, and SRMR = 0.002. The scale’s internal consistency reliability (McDonalds’ omega, ω) was 0.91.

#### Teacher self-efficacy

This study adopted the TALIS2013 teacher self-efficacy scale (OECD, 2014) to assess the participants’ level of teacher self-efficacy. It consisted of 12 items: 4 items for efficacy in instruction (e.g., *Provide an alternative explanation for example when students are confused*); 4 items for efficacy in classroom management (e.g., *Control disruptive behavior in the classroom*); and 4 items for efficacy in student engagement (e.g., *Get students to believe they can do well in school work*). Items were scored using a 5-point Likert scale (1 = strongly disagree to 5 = strongly agree) with higher total scores indicating higher levels of teacher self-efficacy. The goodness of fit is acceptable: χ^2^(48, 14,121) = 3377.273, *p* < 0.001, RMSEA = 0.07, CFI = 0.98, TLI = 0.97, and SRMR = 0.02. The scale’s internal consistency reliability (McDonalds’ omega, ω) was 0.96.

#### Teaching experience and demographic information

Demographic information included information from individual level, which were participant’s age, gender, education level, and professional title, and school level, which was school location. The item to investigate teachers’ teaching experience was “how many years has each participant been a teacher?” and teachers responded to their years of teaching individually.

Individual-level and school-level indicators can affect teacher self-efficacy as well (Avalos, 2011). Therefore, gender, teachers’ educational level, school level, and school location were treated as covariate variables controlled in this study.

### Statistical analyses

**FIGURE 2:**
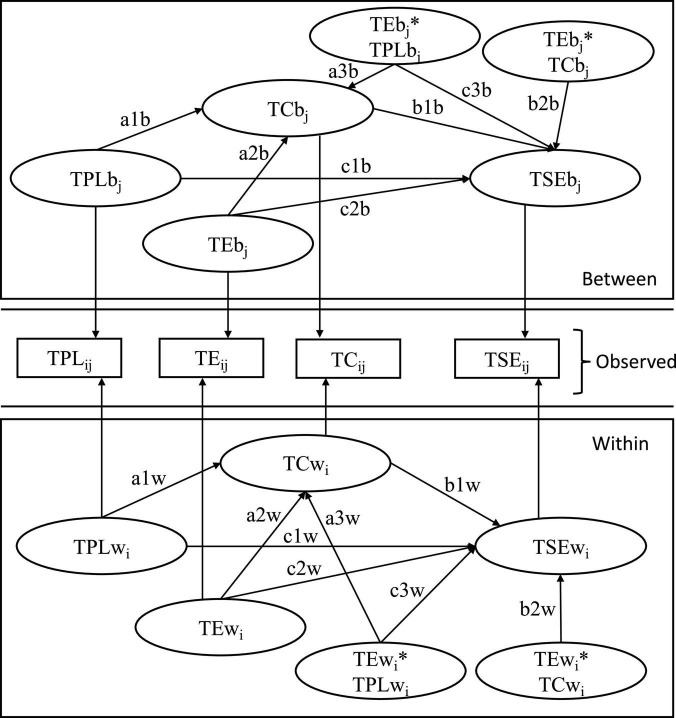
The multilevel structural equation model depicts a 1–1-1 multilevel moderated mediation model. The figure is based on Preacher et al. (2010, 2016). TPL, Teacher-perceived principal leadership; TC, Teacher collaboration; TE, Teaching experience; TSE, Teacher self-efficacy.

The statistical analysis of this study consisted of three steps: first, we checked the descriptive statistics and zero-order correlation between variables. Second, given the hierarchical data structure (teachers nested in schools), we used *Mplus version 8.3* (Muthén and Muthén, 1998–2018) and followed the procedure of Preacher et al. (2010, 2016) to test hypotheses 1–3 using the multilevel structural equation model (MSEM). The multilevel solution allows the variance of level 1 variables to be decomposed into components within and between components and takes into account the fact that the relationships between within and between groups may be different. Because all three variables were evaluated at the teacher level, this model can be described as a 1-1-1 multilevel mediation model. Sobel’s test was used to test the mediating effects (Sobel, 1982). Third, based on the multilevel mediation model, we included the moderating variables teaching experience to build a 1-1-1 multilevel moderating mediation model. The covariate variables at the teacher level were gender and teachers’ education level, and at the school level were school location and school level (see Figure 2). All continuous variables are centralized, and the interaction terms were calculated according to these centralized scores. Moreover, this study analyzed the threshold of teaching experience in terms of the relationship with teaching self-efficacy through the method of segmented regression models (Muggeo, 2008), and analyzed the difference in moderating effect between teachers whose teaching experience is below the threshold (coded as 0) and teachers whose teaching experience is above the threshold (coded as 1).

To ensure the validity of this study, we conducted one of the most widely adopted techniques, namely Harman’s single-factor method, to test for common method bias (Chang et al., 2010; Podsakoff et al., 2013; Tehseen et al., 2017). Exploratory factor analysis shows that the interpretation rate of the first of the three factors was less than 40% (Kong et al., 2020), indicating that the common method bias had little impact on this study.

## Results

### Preliminary analysis

The mean, standard deviation, and zero-order correlation of all variables are shown in Table 1. Teacher self-efficacy was positively related to teacher-perceived principal leadership (*r* = 0.42, *p* < 0.001), teacher collaboration (*r* = 0.46, *p* < 0.001), and teachers’ teaching experience (*r* = 0.09, *p* < 0.001). Therefore, Hypothesis 1 was verified. Teacher-perceived principal leadership was positively related to teacher collaboration (*r* = 0.21, *p* < 0.001) and teachers’ teaching experience (*r* = 0.12, *p* < 0.001). But teacher collaboration was negatively correlated with teachers’ teaching experience (*r* = −0.04, *p* < 0.001).

**TABLE 1:** Descriptive statistics and correlations of the main study variables.

Variables	Mean	*SD*	1	2	3
1. TSE	4.28	0.68			
2. TPL	4.04	0.97	0.42***		
3. TC	4.49	0.84	0.46***	0.21***	
4. TE	14.42	7.41	0.09***	0.12***	−0.04***

***p < 0.001.

### Multilevel mediation model test

In Hypothesis 2, we predicted that teacher collaboration mediated the relationship between teacher-perceived principal leadership and teacher self-efficacy. Given the hierarchical data structure (teachers nested in schools), the group correlation coefficient (ICC) of teacher self-efficacy was 0.10, which was higher than the critical value of 0.059 (Cohen, 1988), we used a multilevel mediation model to analyze the relationship between teacher-perceived principal leadership and teacher self-efficacy at within-group and between-group levels after controlling the covariate variables. Results of this multilevel mediation model investigating the effect of teacher-perceived principal leadership on teacher self-efficacy mediated by teacher collaboration are shown in Table 2. At the within-group level, teacher-perceived principal leadership was significantly related to teacher collaboration (β = 0.169, *p* < 0.001) and teacher self-efficacy (β = 0.231, *p* < 0.001). Further, the relationship between teacher collaboration and teacher self-efficacy was also significant (β = 0.298, *p* < 0.001). The within indirect effect through the teacher collaboration on the relationship between teacher-perceived principal leadership and teacher self-efficacy was significant (β = 0.050, 95% CI [0.035, 0.067], *p* < 0.001). At the between-group level, teacher-perceived principal leadership was significantly related to teacher collaboration (β = 0.207, *p* < 0.001), and was also significantly related to teacher self-efficacy (β = 0.250, *p* < 0.001). The relationship between teacher collaboration and teacher self-efficacy was significant (β = 0.569, *p* < 0.001). It indicated that the within indirect effect through teacher collaboration on the relationship between teacher-perceived principal leadership and teacher self-efficacy was significant (β = 0.118, 95% CI [0.015, 0.220], *p* < 0.001).

**TABLE 2:** Coefficients for the multilevel mediation model predicting teacher self-efficacy.

Variables	Estimate	SE
**Level 1: Within-teacher level**		
Path a1w: TPL → C	0.169***	0.017
Path b1w: TC → TSE	0.298***	0.014
Path c1w: TPL→ TSE	0.231***	0.012
Indirect effect	0.050***	0.007
Residual variance TC	0.587***	0.026
Residual variance TSE	0.295***	0.008
**Level 2: Between-teacher level**		
Intercept	1.812***	0.326
Path a1b: TPL → C	0.207***	0.060
Path b1b: TC→ TSE	0.569***	0.083
Path c1b: TPL → TSE	0.250***	0.033
Indirect effect	0.118**	0.040
Residual variance TC	0.034***	0.007
Residual variance TPL	0.009***	0.001

**p < 0.01, ***p < 0.001.

### Multilevel moderated mediation model test

In Hypothesis 3, this study assumed that teachers’ teaching experience would mediate the indirect relationship between teacher-perceived principal leadership and teacher self-efficacy. The results of the multilevel moderated mediation model are given in Table 3. At the within-group level, the interaction between teacher-perceived principal leadership and teaching experience on teacher collaboration (β = −0.096, *p* < 0.001) and the interaction between teacher-perceived principal leadership and teaching experience on teacher self-efficacy (β = −0.060, *p* < 0.001) were both statistically significant. The average effect size of moderate tests published in major journals was only0.094 (Aguinis et al., 2005), so the current effect at the within-group level was medium. In addition, at the between-group level, the interaction between teacher-perceived principal leadership and teaching experience on teacher collaboration (β = −1.781, *p* < 0.001) was also significant.

**TABLE 3:** Coefficients for the multilevel moderated mediation model predicting teacher self-efficacy.

Variables	Estimate	SE
**Level 1: Within-teacher level**		
Path a1w: TPL → C	0.200***	0.018
Path a2w: TEw → C	0.094***	0.014
Path a3w: TPL*TE → C	−0.096***	0.011
Path b1w: TC → TSE	0.360***	0.016
Path b2w: TC*TE→ TSE	–0.002	0.010
Path c1w: TPL→ TSE	0.332***	0.016
Path c2w: TE→ TSE	0.051***	0.011
Path c3w: TPL*TE→ TSE	−0.060***	0.014
Indirect effect	0.072***	0.009
Residual variance TC	0.827***	0.036
Residual variance TSE	0.630***	0.017
**Level 2: Between-teacher level**		
Intercept	0.910***	0.297
Path a1b: TPL → C	0.303***	0.055
Path a2b: TEb → C	0.601***	0.155
Path a3b: TPL*TE → C	−1.781***	0.297
Path b1b: TC → TSE	0.744***	0.155
Path b2b: TC*TE→ TSE	–1.608	2.176
Path c1b: TPL→ TSE	0.001	0.475
Path c2b: TE→ TSE	–0.931	0.989
Path c3b: TPL*TE→ TSE	3.884	5.069
Indirect effect	0.225***	0.070
Residual variance TC	0.023***	0.005
Residual variance TSE	0.016***	0.009

***p < 0.001. For brevity, the effects of other background variables (gender, education level, school level, school location) were not presented. Std. Coef., Standardized beta coefficients.

Furthermore, we analyzed the segmented regression between teaching experience and teacher self-efficacy and found that there was a breakpoint (β = 2.214, *SE* = 0.052). It indicated that with the increase in teachers’ teaching experience, there existed a threshold in the development of teacher self-efficacy between 2 and 3 years. We took year 3 as the threshold. The segmented regression model results are presented in Table 4, the slope of piecewise function 1 (β = 0.637, 95% CI [0.510, 0.763], *p* < 0.001) and the slope of piecewise function 2 (β = 0.005, 95% CI [0.003,0.007], *p* < 0.001) were different. We recorded the teaching experience below the threshold as L = 0 and the teaching experience above the threshold as H = 1. Then, we calculated the indirect effect of teacher-perceived principal leadership and teacher self-efficacy = (a1_*i*_ + a3_*i*_ * L or H) * b1_*i*_, where, *i* = within-group level (w) or between-group level (b), L = 0, H = 1.

**TABLE 4:** Coefficients of the segmented regression models.

	Estimate	Std. error	*t*-value	95% CI
β_*1*_	0.637***	0.064	9.89	[0.510, 0.763]
β_*2*_	0.005***	0.001	5.72	[0.003, 0.007]

***p < 0.001.

**FIGURE 3:**
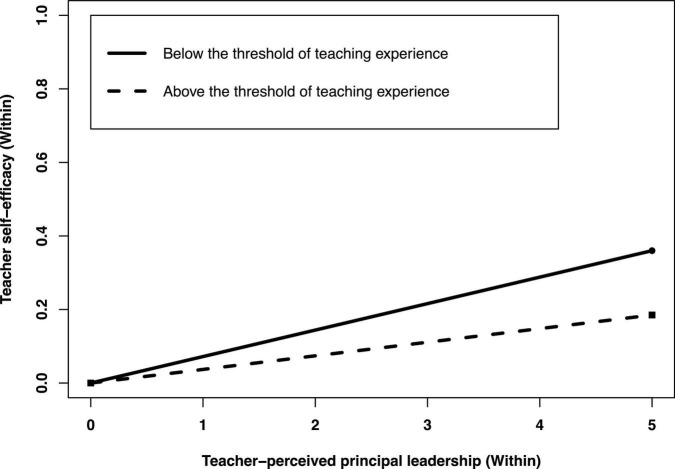
Teaching experience as a moderator of the indirect effect between teacher-perceived principal leadership and teacher self-efficacy through teacher collaboration at the within-group level. Functions are graphed for two levels of positive coping: Teaching experience below and above a threshold.

At the within-group level, to illustrate the moderating effect of teachers’ teaching experience on the indirect effects of teacher-perceived principal leadership on teacher self-efficacy through teacher collaboration, we plotted the regression of teacher-perceived principal leadership on teacher self-efficacy for teaching experience both below and above the threshold. As shown in Figure 3, the simple slope tests showed that the within indirect effect between teachers-perceived principal leadership and teacher self-efficacy was stronger for teaching experience below the threshold (b_*simple*_ = 0.072, *p* < 0.001) than that for above threshold (b_*simple*_ = 0.037, *p* < 0.001).

**FIGURE 4:**
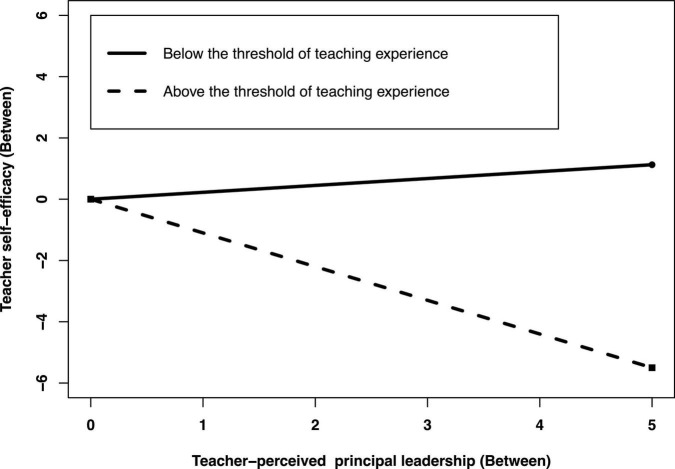
Teaching experience as a moderator of the indirect effect between teacher-perceived principal leadership and teacher self-efficacy through teacher collaboration at the between-group level. Functions are graphed for two levels of positive coping: Teaching experience below and above a threshold.

In addition, at the between-group level, we plotted the regression of teacher-perceived principal leadership on teacher self-efficacy for teaching experience below and above thresholds to illustrate the moderating effect of teachers’ teaching experience on the indirect effects of teacher-perceived principal leadership on teacher self-efficacy through teacher collaboration. As shown in Figure 4, the simple slope test showed that the indirect between effect between teachers-perceived principal leadership and teacher self-efficacy was stronger for teaching experience below threshold (b_*simple*_ = 0.225, *p* < 0.001) than that for above threshold (b_*simple*_ = −1.100, *p* < 0.001).

## Discussion

The effect of principal leadership on teacher self-efficacy has garnered considerable empirical support (Bellibas and Liu, 2017; Prochazka et al., 2017; Gkolia et al., 2018; Liu and Hallinger, 2018; Salanova et al., 2020). However, few previous studies have examined the mediating effect of teachers’ collaboration and moderating effect of years’ teaching experience between teacher-perceived principal leadership and teacher self-efficacy separately with a threshold approach. The linear relationship differs with a cut-off value for the number of years of teaching for teachers. In view of the impact of teacher collaboration and teaching experience, it is necessary to understand the driving force of the development of teacher self-efficacy development more comprehensively. This study has developed a multilevel moderated mediating model to examine the indirect relationship between teacher-perceived principal leadership and teacher self-efficacy through teacher collaboration and tested whether this indirect association was moderated by teaching experience. Furthermore, we explored the difference in the indirect effect of teaching experience below and above the threshold. The main contribution of this study was to improve our understanding of how and when teacher-perceived principal leadership was related to teacher self-efficacy. It provided a broad perspective for the intervention and improvement of teacher self-efficacy.

### Mediating role of teacher collaboration

As hypothesized, this study confirmed the mediating role of teacher collaboration in the multilevel association between teacher-perceived principal leadership and teacher self-efficacy. According to self-efficacy theory (Bandura, 1986, 1997), teacher professional learning activities affect teacher self-efficacy. Some studies have found that teacher self-efficacy is closely related to teacher collaboration (Chong and Kong, 2012; Liu et al., 2021). Other studies have found that principal leadership is closely related to teacher collaboration (Egodawatte et al., 2011; Vangrieken et al., 2015). However, this study makes a novel contribution by examining the mediating role of teacher collaboration between teacher-perceived principal leadership and teacher self-efficacy. Therefore, teacher collaboration was an important mechanism to connect teacher-perceived principal leadership with teacher self-efficacy. To our knowledge, this study was the first to report such results in Confucian culture. These findings illustrated how modern educational management and teacher collaboration affect the development of teacher self-efficacy. Whether within or between teacher groups, teacher collaboration explained and mediated the impact of teacher-perceived principal leadership on teacher self-efficacy. We noted that the indirect impact of teacher collaboration in the teacher group was not particularly strong in this study. One possible reason is that the impact of teacher collaboration is slow and may reflect longitudinally, which might be not fully reflected in this cross-sectional study. The proportion of important mediators identified in the longitudinal study was actually higher than that in the cross-sectional study (Davies et al., 2016). The relatively small effect also suggested that other important mediating factors, such as teacher–peer relationship, should be examined in future research.

Mastery experiences and vicarious experiences in teaching activities are significant factors impacting the development of teacher self-efficacy (Bandura, 1986). Moreover, collective efficacy belief is “shared beliefs in group capacities to organize and execute the courses of action required to produce given attainments” (Bandura, 1997, p. 447). Research conducted in organizations showed that when individuals cooperate, they may share beliefs and attitudes, thus showing similar persuasion and personal standards of conduct (George, 1990, 1996). This study discussed the mediating role of teacher collaboration in the relationship between teacher-perceived principal leadership and teacher self-efficacy. It is an important extension and complement of the current self-efficacy theory. The current research suggested that school leaders should encourage teachers to participate in teacher collaboration, which can improve teacher self-efficacy and promote their teaching and development (deVries et al., 2013; Coldwell, 2017).

### Moderating role of teaching experience

As another important aspect of process-oriented research, Hypothesis 3 assumed that teachers’ teaching experience would moderate the direct or indirect relationship between teacher-perceived principal leadership and teacher self-efficacy. The results showed that within the teacher group, teachers’ teaching experience moderates the path among teacher-perceived principal leadership, teacher collaboration, and teacher self-efficacy. At the between-group level, teachers’ teaching experience moderated the path between teacher-perceived principal leadership and teacher collaboration. Another contribution of this study was to consider the threshold factors of teaching experience and teacher self-efficacy development. This study found that the threshold of teaching effectiveness between novice teachers and experienced teachers is in the 3rd year of teachers’ employment, which provides large-scale investigation evidence for the previous studies (Tschannen-Moran and Hoy, 2007; Fantilli and McDougall, 2009).

Specifically, we have found that both within and between groups, the impact of teacher-perceived principal leadership on teacher collaboration was stronger for teachers below the threshold of teaching experience than for those with teaching experience above the threshold. The prediction ability of teaching experience is consistent with previous studies regarding the relationship between years of teaching experience and teacher self-efficacy (Klassen and Chiu, 2010; Lee et al., 2013; Li et al., 2019).

Results of the above model showed that novice teachers whose teaching years were below the threshold were more likely to benefit from self-efficacy renewal, collaborative activities, and reflection. Participation in teacher collaboration can meet the needs of young teachers in improving their practice, such as classroom management (Grangeat and Gray, 2007; deVries et al., 2013). For them, emotional support, information support, positive interaction with tutors, and resource integration are the four key factors affecting their success adjustment and promotion in school (Li et al., 2019). An emphasis has also been on the value of teacher collaboration for experienced teachers, and their self-efficacy is related to more participation in reflective and collaborative activities. They may not be able to experience mastery in update activities, because they have higher abilities and accumulated diversified knowledge to solve potential problems (Eros, 2011; Louws et al., 2017).

## Limitations

Several limitations must be considered when interpreting the results of this study. First, the cross-sectional design of this study excludes the test of causality or directionality. Although the current research showed that teacher-perceived principal leadership may improve the development of teacher self-efficacy, the increase of teacher self-efficacy may also improve teacher-perceived principal leadership. Future studies can use experiments or longitudinal designs to clarify the causality of these variables. Second, this study mainly relied on teachers’ self-report to collect data. Future research should collect data from multiple insiders (e.g., principals, students, parents, or peers). Finally, the participants in this study were only middle-school teachers in Shandong Province, China, rather than all teachers from different locations. Therefore, caution should be exercised in applying the research results to groups from other cultures. Future research can be conducted in other samples (e.g., a sample of primary school teachers or teachers from other cultural backgrounds) to test the model.

## Conclusion

Despite these limitations, results still have important practical significance. First, the results emphasized the importance of principal leadership in the development of teacher self-efficacy and showed that teacher-perceived principal leadership may be directly or indirectly related to teacher self-efficacy. School administrators could improve teachers’ participation to enhance teacher self-efficacy through teacher collaboration and communication among teachers. Second, this study found that the threshold for teaching experience regarding the development of teacher self-efficacy is approximately in year 3. Compared with experienced teachers whose teaching experience was above the threshold, novice teachers’ participation in teacher collaboration benefited more in the development of teacher self-efficacy. This conclusion reminded educational practitioners to pay more attention to the professional development of novice teachers, and it will be more effective to develop teacher self-efficacy for teachers whose teaching experience is below the threshold of teaching years.

To conclude, this study contributed to the literature by examining a multilevel moderated mediation model, which provided a unique perspective for understanding the relationship between teacher-perceived principal leadership and teacher self-efficacy. It provided evidence that the relationship between teacher-perceived principal leadership and teacher self-efficacy was partly mediated by teacher collaboration. In addition, it showed the existence of a threshold for teaching experience for teacher self-efficacy development. These findings were in an aim to promote the current understanding of the mechanism regarding the relationship between teacher-perceived principal leadership and teacher self-efficacy.

## Data availability statement

The raw data supporting the conclusions of this article will be made available by the authors, without undue reservation.

## Ethics statement

Ethical review and approval was not required for the study on human participants in accordance with the local legislation and institutional requirements. Written informed consent from the patients/participants or patients/participants legal guardian/next of kin was not required to participate in this study in accordance with the national legislation and the institutional requirements.

## Author contributions

ZX: conceptualization, methodology, formal analysis, funding acquisition, writing – original draft, writing – review and editing, visualization, and validation. RW: conceptualization, writing – review and editing. JL and HL: investigation, data curation, resources, supervision, and project administration. All authors contributed to the article and approved the submitted version.
